# Long-term physical health consequences of abortion in Taiwan, 2000 to 2013: A nationwide retrospective cohort study

**DOI:** 10.1097/MD.0000000000011785

**Published:** 2018-08-03

**Authors:** Tsai-Bei Lin, Men-Fong Hsieh, Ying-Chung Hou, Yu-Ling Hsueh, Hui-Ping Chang, Yuan-Tsung Tseng

**Affiliations:** a Department of Obstetrics and Gynecology; b Department of Family Medicine; c Department of Traditional Chinese Medicine; d Department of Medical Research, Tainan Municipal Hospital(Managed by Show Chwan Medical Care Corporation), Tainan, Taiwan (R.O.C.).

**Keywords:** induced abortion, infertility, NHIRD, spontaneous abortion

## Abstract

The aim of this study was to quantitatively estimate the long-term risk of abortion-related consequences and comorbidities.

We identified 36,375 patients with at least 2 diagnosed abortions from 2000 to 2013 and included them in the abortion group. This group was further subdivided into 4 subgroups: spontaneous abortion, induced abortion, nonspecific abortion, and mixed-type abortion groups. For comparison, another 36,375 pregnant women from the National Health Insurance Research Database of Taiwan were included in the nonabortion group. For the puerperal cohort, the index year was defined as the year with the occurrence of at least 1 pregnancy. The puerperal cohort was then matched to the abortion cohort by age; comorbidities of diabetes mellitus, hypertension, and hyperlipidemia; and index year at a 1:1 ratio. The data of these cohorts were used to examine the risk of abortion-related consequences and comorbidities in pregnant women after a mean follow-up period of 7.60 person-years.

The spontaneous abortion group exhibited significantly elevated adjusted hazard ratios (HRs) of 1.493 for pelvic inflammatory disease (*P* < .001), 1.680 for urinary tract infection (*P* < .001), 3.771 for ectopic pregnancy (*P* < .001), and 1.938 for infertility with no subsequent conception (*P* < .001). However, this group exhibited statistically insignificant HRs of 1.709 for placenta previa (*P* = .260), 2.982 for placenta abruption (*P* = .344), 1.499 for incompetent cervix (*P* = .658), and 0.854 for early onset of labor (*P* = .624). The induced abortion group showed a statistically significant elevated adjusted HR of 1.291 for urinary tract infection (*P* = .008) but statistically insignificant HRs of 1.031 for pelvic inflammatory disease, 1.637 for ectopic pregnancy, 5.114 for placenta previa, 65.434 for placenta abruption, 0.998 for incompetent cervix, 0.285 for early onset of labor, and 1.019 for subsequent infertility with no subsequent conception.

Clinicians encountering patients in a predicament such as spontaneous or induced abortion should unprejudicely and objectively inform the patients of the effects or influence of abortion on their physical health, including statistically significant and insignificant risks. Induced abortion may not be an independent risk factor for subsequent infertility.

## 1 Introduction

Medically, abortion is defined as pregnancy termination before 20 weeks of gestation or birthweight less than 500 g; it includes spontaneous, threatened, inevitable, complete, incomplete, missed, recurrent, and induced abortions (IAs), and pregnancy may be terminated either medically or surgically.^[1]^ For most of the 20th century, the term “miscarriage” has been used instead of spontaneous abortion (SA) because this terminology is more patient-centric. In this study, abortion refers to both IAs and SAs.^[2]^

Globally, unsafe termination of pregnancy remains a major public health problem, and the World Health Organization has reported that 21.6 million unsafe abortions occurred in 2008.^[3]^ Abortion encompasses a wide spectrum of tragedies. Approximately 1 in 3 women will have at least 1 pregnancy termination. Some studies have evaluated the short-term complications of unsafe abortion in low-income countries.^[2,4]^ Another study investigated IA and its related consequences.^[5]^ Data on overall maternal health and the subsequent pregnancy outcome after abortion are limited. In addition, abortion is an exposure that cannot be assigned to women by chance as part of an experimental design.^[5]^ Therefore, the long-term consequence of abortion may not have been comprehensively observed and estimated.

In Taiwan, abortion was legalized in 1985, and the National Health Insurance Administration Ministry of Health and Welfare was set up in 1995.^[6]^ Appropriate training, convenience, technology, and equitable access to safe termination services have been provided through health care facilities. In this retrospective cohort study, the nonabortion group was matched to the abortion group by age, sex, and index date at a 1:1 ratio and long-term follow-up was conducted to estimate the adjusted HR of abortion-related consequences in a safe environment. A population-based cohort was employed, and we investigated the long-term risk of abortion-related consequences and comorbidities.

## 2 Materials and methods

### 2.1 Data sources

**Figure 1:**
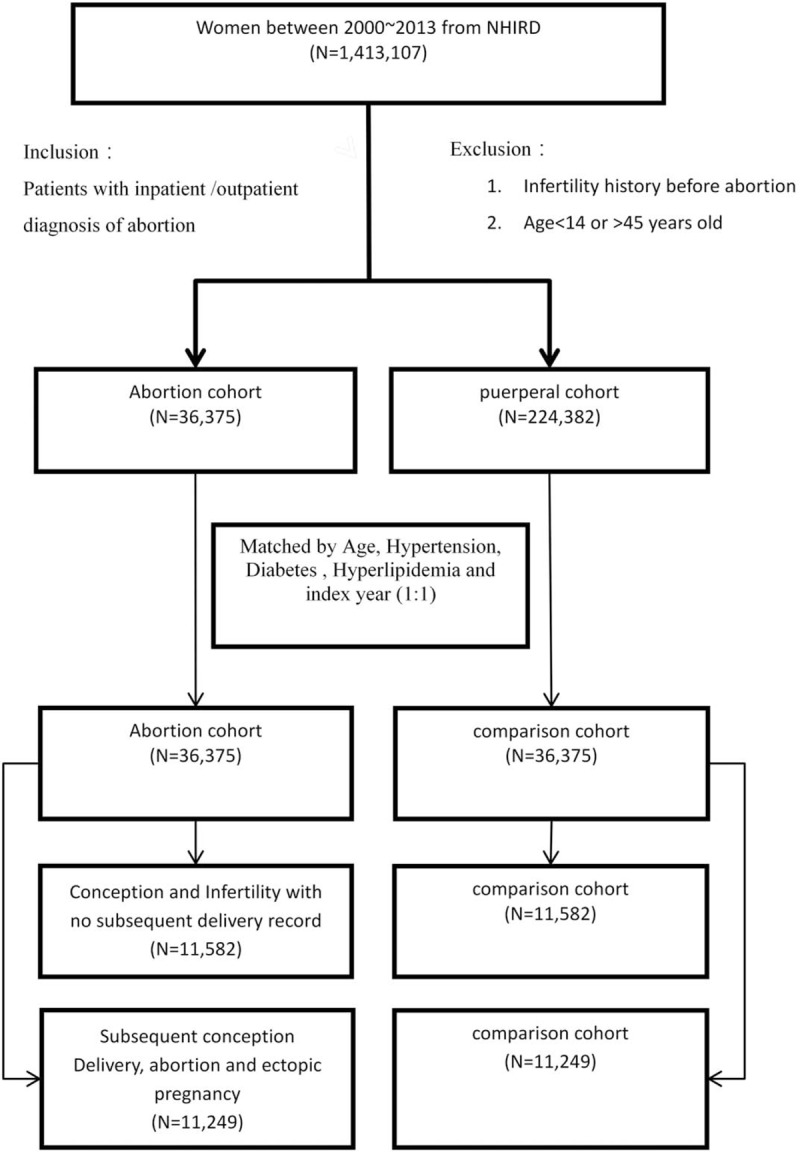
Data process flow. The study population was identified from a nationwide cohort of 1,413,107 women from January 1, 2000, to December 31, 2013. The comparison group (n = 36,375) was matched to the abortion group (n = 36,375) by age, hypertension, diabetes, hyperlipidemia, and index year with propensity score matching.

We conducted a nationwide cohort study using the National Health Insurance Research Database (NHIRD) of Taiwan and recruited women with confirmed diagnosis of abortion. The patients were selected from the NHIRD. This database contains all medical claims data of 1,413,107 women who were randomly sampled from 23 million insurants of the NHI program. The database has a large sample size and provided us with an excellent opportunity to study the risks to patients from abortion. The selection criteria and enrolment of the study cohort are displayed in Fig. 1. Initially, 36,375 patients with at least 2 abortions and no delivery record within 9 months were identified. After applying the inclusion and exclusion criteria and comparing the abortion and the nonabortion groups, 36,375 nonabortion patients were included in the study and were matched to patients with abortions by age, sex, and index year at a 1:1 ratio. Our final study cohort included 36,375 women in the study cohort and 36,375 women in the comparison cohort.

### 2.2 Study participants

We identified all patients with abortions between January 1, 2000, and December 31, 2013, from the NHIRD by using International Classification of Diseases, Ninth Revision, Clinical Modification (ICD-9-CM) codes 634–637. Patients with at least 2 abortions and no delivery record within 9 months were included in our study (Fig. 1). The index date was defined as the date on which the patient was admitted for abortion. Information on patients’ baseline characteristics and comorbidities, including previous hypertension, diabetes, and hyperlipidemia, was collected. Patients were followed up from the index date to the date of abortion, loss to follow-up, or December 31, 2013.

The comparison cohort was selected from the puerperal cohort. Participants were then matched by age; comorbidities of diabetes mellitus (DM), hypertension, and hyperlipidemia; and index year at a 1:1 ratio. For the puerperal cohort, the index year was defined as the year with the occurrence of at least 1 pregnancy. The puerperal cohort was then matched to the abortion cohort by age; comorbidities of DM, hypertension, and hyperlipidemia; and index year in a 1:1 ratio. The end of the follow-up period for analysis was marked on the day of comorbidity diagnosis, withdrawal from the NHI program, death, or the end of this study. To ensure that only newly diagnosed cases were detected for the endpoint, we excluded patients with comorbidity diagnosis in the 365-day period before the index date of abortion for both cohorts.

### 2.3 Exclusion criteria

Patients aged < 14 years or > 45 years were first excluded. Subsequently, patients with newly diagnosed abortion, which was defined as abortion diagnosed on the index date, were excluded to ensure consistency in disease severity and duration among patients with abortion. To avoid the carryover effect, we excluded patients who received abortion before the index date and those who had a delivery < 9 months after the index date.

### 2.4 Study outcomes and covariate measurements

The Institutional Review Board of Show-Chwan Memorial Hospital approved this study and granted a waiver of informed consent from participants on April 19, 2016 (SCMH_IRB NO:1050306). Baseline comorbidities were identified using ICD-9-CM diagnosis codes, and the operation strategies of delivery after the index date were also evaluated. The primary outcome was the composite event of subsequent infertility. On the basis of the ICD-9 diagnosis code of 628.x, subsequent infertility was defined as no record of pregnancy after abortion. All comorbidities were identified on the basis of the data from the NHIRD. Other secondary outcomes were pelvic inflammatory disease, urinary tract infection (UTI), placenta previa, premature separation of placenta, cervical incompetence, early onset of labor, ectopic pregnancy, ischemic heart disease, atherosclerosis, heart failure, arrhythmia, breast cancer, cervical cancer, uterine cancer, and ovarian cancer.

### 2.5 Analytic variables and definitions

The observation period was from the index date to the end of 2013, death, or the development of outcomes, whichever occurred first. As described previously, we estimated the incidence of various diseases by using ICD-9 diagnosis codes in patient records, including abortion (ICD-9 codes 634–637), salpingitis and oophoritis (ICD-9 code 614), inflammatory diseases of uterus and cervicitis (ICD-9 codes 615 and 616), infertility (ICD-9 code 628), UTI (ICD-9 code 599.0), placenta previa (ICD-9 codes 641.0–641.13), premature separation of placenta (ICD-9 codes 641.2–641.23), cervical incompetence (ICD-9 codes 654.50–654.54), early onset of labor (ICD-9 codes 644.2–644.21), ectopic pregnancy (ICD-9 codes 633.0–633.9), ischemic heart disease (ICD-9 codes 410–414), atherosclerosis (ICD-9 code 440), heart failure (ICD-9 code 428), arrhythmia (ICD-9 code 427), breast cancer (ICD-9 code 174), cervical cancer (ICD-9 code 180), uterine cancer (ICD-9 code 182), and ovarian cancer (ICD-9 code 183). Abortion was confirmed in the abortion group by at least 2 claims in outpatient records regarding abortion and no delivery record within 9 months. We extracted all the comorbidities and corresponding treatments starting a year before diagnosis from NHIRD.

### 2.6 Propensity score matching

We identified the comparison cohort by using propensity score matching (PSM). We applied a multivariate logistic regression model to estimate the PS for patients receiving abortion. We then estimated the risk of abortion after PS matching to control for confounding factors and to ensure comparativeness between both abortion and nonabortion groups. Potential confounders and covariates related to the outcome, such as surgery records and comorbidities at baseline, were included in the regression model.

### 2.7 Statistical analyses

To minimize the bias in the estimated effect (group difference) in this study, the abortion cohort was matched to the comparison cohort by patient age, baseline comorbidities, and index date at a 1:1 ratio by using PSM. The PSM algorithm was based on the nearest-neighbor method and used the caliper radius (set at 0), which signifies a tolerance level for the maximum distance in the PS. The matching procedure was performed using R version 3.1 and SPSS version 23.0 (IBM SPSS Inc, Chicago, IL). The clinical characteristics of the abortion and nonabortion groups were compared using the McNemar test for categorical variables and the paired *t* test for continuous variables. Time to the first occurrence of a predefined primary outcome after the index hospitalization was compared between the study groups by using Cox proportional hazard models with adjustment of the PS. All statistical analyses were conducted using SPSS version 23.0 (IBM SPSS Inc, Chicago, IL).

### 2.8 Study patients

We provided 2 sets of statistics to investigate the relationship between abortion risks. A total of 36,375 patients diagnosed with abortion who were at risk of subsequent sequelae from January 1, 2000, to December 31, 2013, were identified and included in our study cohort. The comparison cohort was matched to these 36,375 patients in the abortion group. Among the 36,375 patients in the abortion group, 11,582 patients having conception and subsequent infertility with no delivery record were identified and included in a separate group. Subsequently, 11,582 patients in the comparison group were matched to the patients in the aforementioned group. The mean age of the abortion and comparison cohorts was 34.52 and 34.51 years, respectively. The mean follow-up period of the abortion and comparison cohorts was 7.60 and 7.65 person-years. No differences were observed in baseline characteristics and comorbidities between the abortion and comparison cohorts after PSM (Table 1).

**Table 1:**
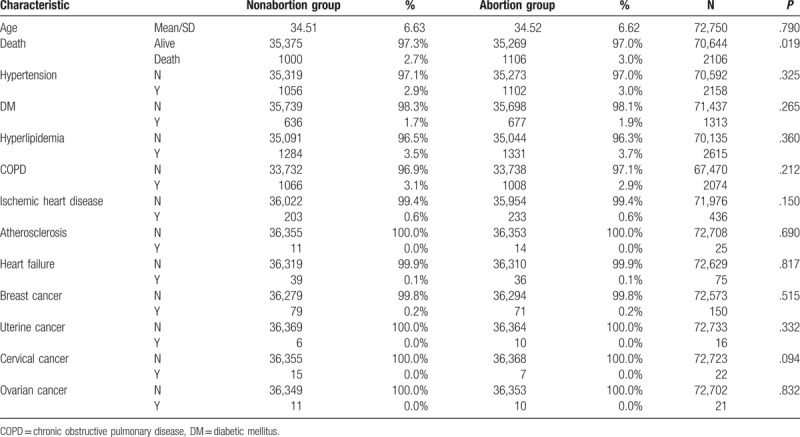
Distribution of selected characteristics and their related comorbidity ratio in abortion and nonabortion groups.

## 3 Results

### 3.1 Cohort characteristics

This study included 36,375 patients with at least 2 diagnosed abortions and no delivery record within 9 months in the abortion group and 36,375 matched women in the nonabortion group. Demographics of the 2 groups are presented in Fig. 1 and Tables 1 to 7. The baseline characteristics were generally balanced after matching.

**Table 2:**
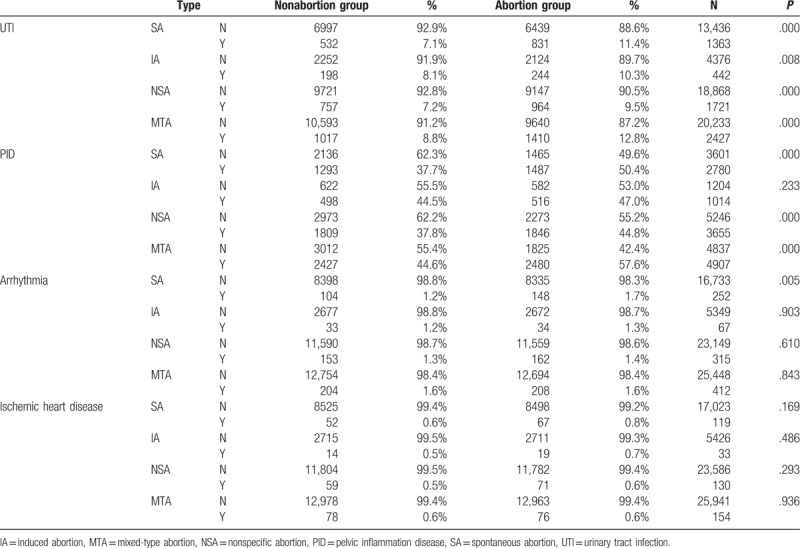
Characteristic ratio of 4 abortion and nonabortion groups and their related problems, including subsequent UTI, PID, arrhythmia, and ischemic heart disease.

**Table 3:**
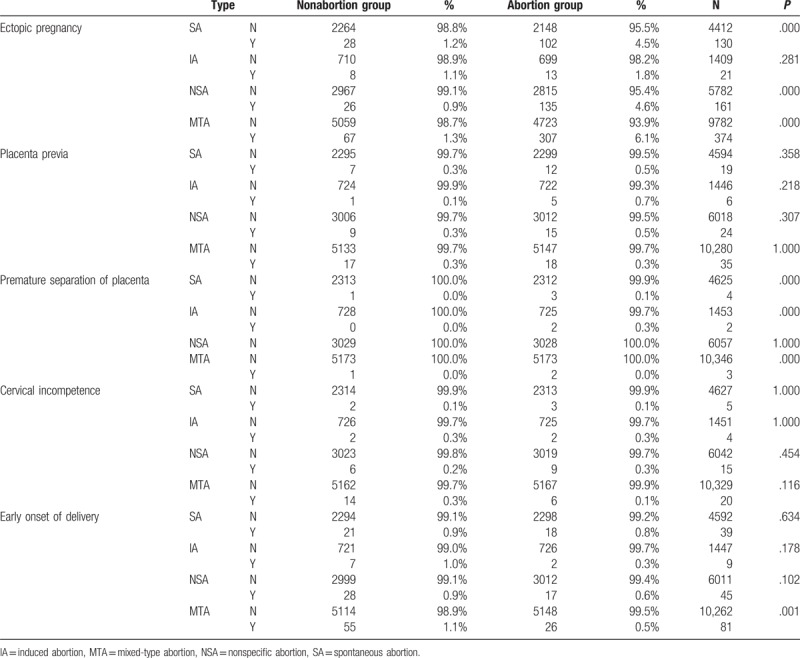
Characteristic ratio of 4 abortion and nonabortion groups and their related obstetric problems.

**Table 4:**
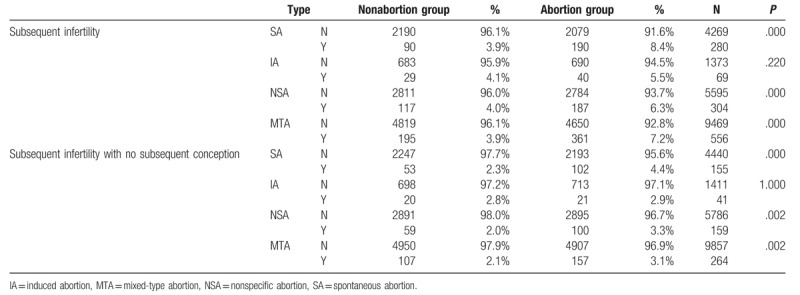
Characteristic ratio of 4 abortion and nonabortion groups and their related problems, including subsequent infertility-related problems.

**Table 5:**
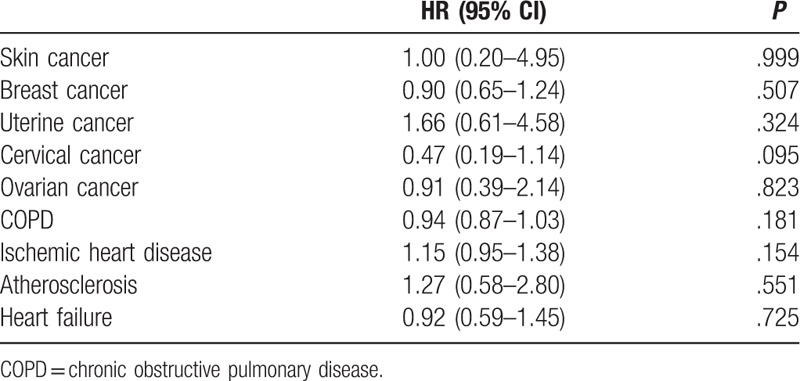
Comparison of HR between abortion and nonabortion groups and their related comorbidities.

**Table 6:**
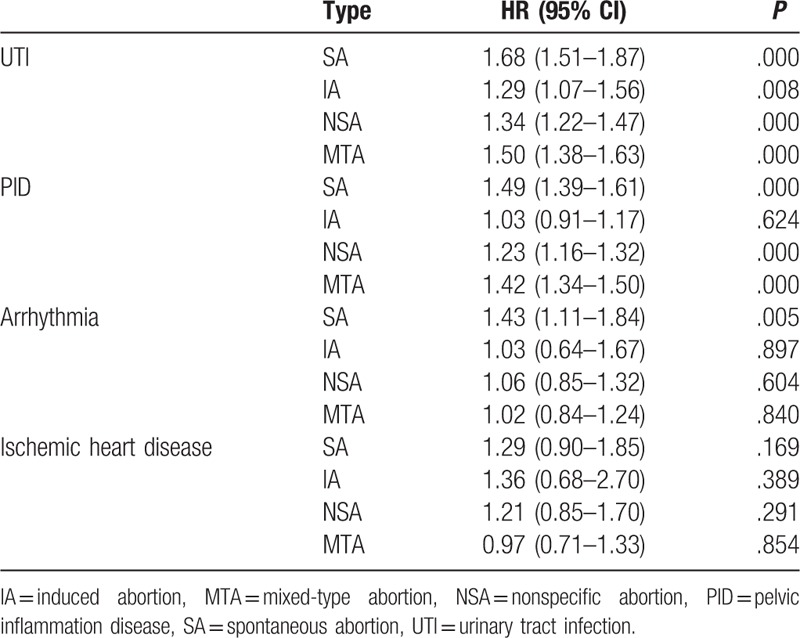
Comparison of HR among 4 abortion and nonabortion groups and their subsequent UTI, PID, arrhythmia, and ischemic heart disease.

**Table 7:**
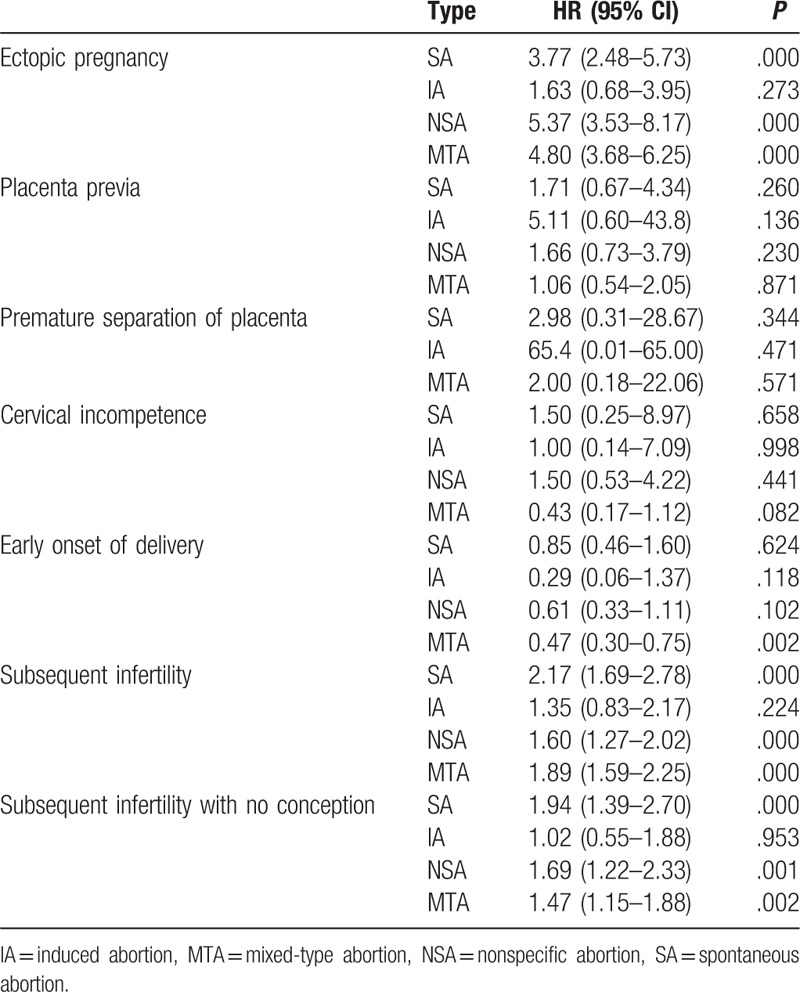
Comparison of HR among 4 abortion and nonabortion groups and their subsequent obstetric and infertility-related problems.

### 3.2 Abortion-related risk of comorbidities in obstetrics and reproduction

In reproductive tract infections (RTIs) and UTIs, the IA group had an HR of 1.291 for UTI (*P* = .008) but an insignificant HR of 1.031 for pelvic inflammatory disease (*P* = .624). The SA group had significantly elevated adjusted HRs of 1.493 for pelvic inflammatory disease (*P* < .001) and 1.680 for UTI (*P* < .001; Tables 2 and 6).

Regarding obstetric problems, the HR for placenta previa was 1.709 in the SA group (*P* = .260) and 5.114 in the IA group (*P* = .136); for premature separation of the placenta was 2.982 in the SA group (*P* = .344) and 65.434 in the IA group (*P* = .471); for cervical incompetence was 1.449 in the SA group (*P* = .658) and 0.998 in the IA group (*P* = .998); and for early onset of labor was 0.854 in the SA group (*P* = .624) and 0.285 in the IA group (*P* = .118).

Regarding ectopic pregnancy, the SA group had a significantly elevated HR of 3.771 (*P* < .001) for ectopic pregnancy in the subsequent pregnancy. The IA group had an HR of 1.637 (*P* = .273) for ectopic pregnancy (Tables 3 and 7).

Regarding reproductive tract and breast tumors (RTTs), the HRs for breast cancer (0.897; *P* = .507), cervical cancer (0.465; *P* = .095), uterine cancer (1.663; *P* = .324), and ovarian cancer (0.907; *P* = .823) in the abortion group were nonsignificant. Thus, abortion may not be associated with RTTs and breast tumors (Table 5).

Regarding abortion-related comorbidities, the HRs for chronic obstructive pulmonary disease (0.94; *P* = .181), ischemic heart disease (1.15; *P* = .154), atherosclerosis (1.27; *P* = .551), heart failure (0.92, *P* = .725) in the abortion group were nonsignificant. The HRs for arrhythmia (1.43, *P* = .005) in the SA group were significant (Tables 5 and 6).

### 3.3 Abortion and risk of subsequent infertility

In our abortion group, SA after the index date significantly increased the risk of subsequent infertility [crude HR, 1.83; 95% confidence interval (95% CI), 1.63–2.06; ICD-9 codes 635 and 636]. A Kaplan–Meier curve was used to compare the cumulative incidences of abortion across age, sex, and index date in the abortion and nonabortion groups. This curve showed a higher cumulative incidence of subsequent infertility in the abortion group (Fig. 1 and Tables 4 and 7). The multivariable Cox proportional model was used to calculate adjusted HRs for the incidence of infertility, and the result is summarized in Tables 4 and 7. Further observation of the IA group with subsequent infertility and no delivery record showed that the IA may not be an independent risk factor for infertility (adjusted hazard ratio, 1.02; 95% CI, 0.55–1.88; ICD-9 codes 635 and 636).

## 4 Discussion

### 4.1 Decision of whether to continue or terminate a pregnancy

Studying the long-term health effects of abortion is difficult. Such a study is not only associated with experimental basis but also associated with the challenging problem of assigning an appropriate comparison group. In addition, regret, remorse, or shame may cause women not to disclose having made such a decision (abortion) when queried about their reproductive histories. Therefore, pregnant women are more objective for a comparison group than those selected based on their reproductive histories.^[5]^ Thus, in this study, for estimating the risk of abortion-related consequences and comorbidities, the comparison group only included women with the pregnancy and childbirth occurrence in the same year and no abortion record in the study period. The study findings may also serve as a reference for women faced with making difficult decisions about whether to continue or terminate a pregnancy and those who have to accept SA.

### 4.2 Abortion-related reproductive tract infections and UTIs

After abortion, women are at an increased risk of RTIs. In high-income countries, IA is associated with a 5% to 10% higher risk of postabortion infection, including pelvic inflammation disease (PID).^[7]^ The risk of iatrogenic infections in relation to the abortion procedure may be assumed to be higher in resource-poor settings, where hospitals may lack appropriate antimicrobial therapy and infection control practices.^[7]^

In our study, the IA group had a significantly elevated HR for UTI but a nonsignificant HR for pelvic inflammatory disease. By contrast, the SA group had a significantly elevated HR for both pelvic inflammatory disease and UTI (Tables 2 and 6).

RTIs and UTIs are associated with an increased risk of iatrogenic infections. Routine administration of prophylactic antibiotics to all women with SA or IA should be considered.

### 4.3 Abortion-related obstetric problems

Henriet and Kaminski^[8]^ found increased odds for very preterm birth in all subgroups: 1.19 after 1 IA, 1.69 after 2 IAs, and 2.78 after 3 IAs, demonstrating a dose–response relationship. Increased odds for preterm birth (<37 weeks) and low birthweight (<2500 and < 1500 g) were observed only in mothers with 3 or more IAs: 1.35 after 1 IA, 1.43 after 2 IAs, and 2.25 after 3 IAs.^[8]^

It has been suggested that both infections before and after IA and surgical procedures may be the underlying mechanism for the increased risk of preterm births in subsequent pregnancies.^[8,9]^ However, in the study by Henriet and Kaminski,^[8]^ information on previous IA was collected through interviews. Moreover, in a study by Ancel et al,^[9]^ information on previous IA was collected through interviews during hospitalization in the maternity unit. This might cause a difference in the result, particularly because women may intentionally conceal their abortion history. Women with a history of IA have an increased risk of intra-amniotic infection, intrapartum infection, and infection in their infants. A study found a relationship between previous IA and preterm birth after placenta previa and other maternal hemorrhage.^[9]^ It has been suggested that surgical IA, which can damage the endometrium, may increase faulty placentation, causing preterm delivery in the subsequent pregnancy. Furthermore, surgical IA may cause mechanical trauma to the cervix, increasing the risk of cervical insufficiency.^[9]^

In our study, the HRs for placenta previa, premature separation of the placenta, cervical incompetence, and early onset of labor in the SA and IA groups were nonsignificant in the subsequent pregnancy. Abortion appeared to have little influence on the subsequent pregnancy. SA or IA did not result in a significantly elevated HR in the subsequent pregnancy. However, this study did not include information on the number of abortions and how these abortions occurred. Therefore, additional studies should include the number of abortions and how they occurred to investigate obstetric problems such as those described in Tables 3 and 7.

### 4.4 Ectopic pregnancy

Tharaux-Deneux et al^[10]^ studied women with no previous ectopic pregnancy and showed that, after controlling for the main risk factors for ectopic pregnancy, prior IA is associated with an increased risk of ectopic pregnancy [odds ratio (OR), 1.5; 95% CI, 1.0–2.0]. They believed that under-reporting was possible.^[10]^ The main source of bias was the ascertainment of previous IA, which was self-reported by patients.

In our study, the SA group had a significantly elevated HR for ectopic pregnancy in the subsequent pregnancy. However, the HR for the IA group was nonsignificant. This finding suggests that the SA group had a higher risk of ectopic pregnancy (Tables 3 and 7).

Further studies should investigate why SA is associated with a statistically significant high risk of ectopic pregnancy.

### 4.5 Abortion and subsequent infertility

Clinically, infertility is currently defined as 1 year of unwanted nonconception with unprotected intercourse in the fertile phase of the menstrual cycle.^[11]^ After 48 months of unprotected intercourse without successful pregnancy, approximately 5% of the couples are definitely infertile, with a nearly zero chance of having a spontaneous pregnancy in the future.^[11]^ Subfertility refers to couples who conceive after 12 months of attempted impregnation.^[11]^ Women undergoing abortion are defined as fertile. Thorp et al^[5]^ found that women who have never conceived or those who have given birth to children do not constitute an ideal comparison group. To calculate the real rate of subsequent infertility is extremely difficult because we do not know the actual number of women who want to be pregnant without contraception in all groups.

Thus, we included the number of women who conceived and were diagnosed as being infertile to attempt to obtain the number of women who wanted to get pregnant without contraception. However, we could not determine the real number of women attempting to become pregnant without searching for infertility service in the database. Therefore, the calculated ratio of subsequent infertility in women with abortion should be less than the actual ratio. This may cause the calculated rate to be less than the actual rate because some women with subsequent infertility may not want to seek medical treatment. However, the risk may be reliable for the adjusted control group, which included women experiencing the same situation.

In our study, HR for subsequent infertility in the IA group (HR, 1.346) was statistically insignificantly higher than that in the control group after a mean follow-up period of 7.60 person-years. Moreover, HR for subsequent infertility without subsequent conception was also statistically insignificant in the IA group (HR, 1.019) after a mean follow-up period of 7.60 person-years. IA may not be an independent risk factor for subsequent infertility (Tables 4 and 7).

HR for subsequent infertility without subsequent conception was statistically significant in the SA group (HR, 1.938; *P* < .001) after a mean follow-up period of 7.60 person-years. The underlying mechanism for this trend should be further investigated.

### 4.6 Abortion-related reproductive tract tumor and breast tumor

Potential links between breast cancer and abortion are the most controversial long-term health consequence explored in a review by Thorp et al.^[5]^ Their study demonstrated that the “loss of protection” effect is the most pronounced in women aged less than 20 years who elect to undergo abortion rather than continue their pregnancy.^[5]^ They found a small but statistically significant OR of 1.3.

In our study, the HRs for breast, cervical, uterine, and ovarian cancers in the abortion group were nonsignificant after a mean follow-up period of 7.60 person-years. Thus, abortion may not be associated with RTTs and breast tumors (Tables 1 and 5).

### 4.7 Abortion-related comorbidity

Increasing evidence has demonstrated that women with adverse pregnancy outcomes, including pre-eclampsia, gestational diabetes, or pregnancy-induced hypertension, are at an increased risk of cardiovascular disease. In a meta-analysis of 5,17,504 individuals with 1685 cases, Oliver- Oliver-Williams et al^[12]^ indicated that a history of miscarriage or recurrent miscarriage is associated with high odds of developing coronary heart disease (HR, 1.45; 95% CI, 1.18–1.78). They also reported a strong association between recurrent miscarriage and coronary heart disease (HR, 1.99; 95% CI, 1.13–3.50).^[12,13]^ Wagner et al^[13]^ analyzed 60,105 women and determined HRs for ischemic heart disease in women with 1 miscarriage (HR, 0.82; 95% CI, 0.68–0.98, N = 9419), 2 miscarriages (HR, 1.75; 95% CI, 1.22–2.52, N = 940), or 3 or more miscarriages (HR, 3.18; 95% CI, 1.49–6.80, N = 167).

In our study, the HRs for chronic obstructive pulmonary disease, ischemic heart disease, atherosclerosis, and heart failure in the SA and IA groups were nonsignificant after a mean follow-up period of 7.60 person-years. However, the HR for arrhythmia in the SA group was significant (Tables 1 and 5).

### 4.8 Study limitations

This study has several weaknesses and strengths. First, we identified patients with abortion from a large nationwide population-based database, which is representative of the commercially insured Taiwanese population. To the best of our knowledge, this is the first population-based study to investigate the associated risks in patient with abortion. The period covered by the data in the research database is limited; in this study, the average observation period was 7.6 years. Although the increased incidence in patients with abortion is a novel finding, this finding must be further tested in other cohort studies.

Second, we primarily depended on diagnosis codes to identify patients with abortion, which may cause outcome misclassification. In addition, the treatment and diagnosis codes for abortion, rather than the diagnosis code alone, were used to minimize surveillance bias in our study. This methodology should reduce the possibility of misclassification in a claim-based study.

Third, the NHIRD does not contain information on some potential confounding covariates such as serum uric acid level, personal smoking history, and body mass index. However, we attempted to partially adjust for lifestyle factors by matching the nonabortion group to the abortion group by comorbidities of hypertension, diabetes, and hyperlipidemia.

## 5 Conclusion

Clinicians encountering patients in a predicament such as SA or IA should unprejudicely and objectively inform the patients of the effects or influence of abortion on their physical health, including statistically significant and insignificant risks, even if the risks might have implication according to the moral values of some patients. Except for infection, IA or SA did not result in an increased risk of obstetrics- and reproduction-related morbidities after an average of 7.6 years of observation. IA may not be an independent risk factor for subsequent infertility.

## Author contributions

**Data curation:** Yuan-Tsung Tseng.

**Formal analysis:** Yuan-Tsung Tseng.

**Funding acquisition:** Men-Fong Hsieh.

**Resources:** Ying-Chung Hou, Hui-Ping Chang.

**Supervision:** Men-Fong Hsieh, Yu-Ling Hsueh.

**Writing – original draft:** Tsai-Bei Lin.

Author name: orcid number
